# Comparison of pregnancy incidence among African women in a randomized trial of intramuscular depot medroxyprogesterone acetate (DMPA-IM), a copper intrauterine device (IUDs) or a levonorgestrel (LNG) implant for contraception^☆^^☆☆^

**DOI:** 10.1016/j.conx.2020.100026

**Published:** 2020-05-28

**Authors:** Maricianah Onono, Kavita Nanda, Kate B. Heller, Doug Taylor, Irina Yacobson, Renee Heffron, Margaret Phiri Kasaro, Cheryl E. Louw, Zelda Nhlabasti, Thesla Palanee-Phillips, Jenni Smit, Imelda Wakhungu, Peter B. Gichangi, Nelly R. Mugo, Charles Morrison, Jared M. Baeten, for the Evidence for Contraceptive Options and HIV Outcomes (ECHO) Trial Consortium

**Affiliations:** a Kenya Medical Research Institute Center for Microbiology Research, P.O. Box 19464-00202, Nairobi, Kenya; b FHI 360, Durham, USA; c University of Washington, Seattle, USA; d UNC Global Projects Zambia & University of North Carolina at Chapel Hill, Zambia; e Madibeng Centre for Research, Brits, South Africa; f Department of Family Medicine, University of Pretoria, Pretoria, South Africa; g Family Life Association of eSwatini & ICAP at Columbia University, eSwatini; h Wits Reproductive Health and HIV Institute, University of the Witwatersrand, School of Clinical Medicine, Johannesburg, South Africa; i University of the Witwatersrand, Durban, South Africa; j International Center for Reproductive Health, Kenya; k Technical University of Mombasa, Mombasa, Kenya

**Keywords:** Hormonal contraception, Injectables, Implants, Copper intrauterine device, Africa, Pregnancy incidence

## Abstract

**Objective:**

The objective was to address bias in contraception efficacy studies through a randomized study trial of intramuscular depot medroxyprogesterone acetate (DMPA-IM), a copper intrauterine device (IUDs) and a levonorgestrel (LNG) implant.

**Study design:**

We analyzed data from the Evidence for Contraceptive Options and HIV Outcomes Trial, which assessed HIV incidence among 7829 women from 12 sites in eSwatini, Kenya, South Africa and Zambia seeking effective contraception and who consented to be randomized to DMPA-IM, copper IUD or LNG implant. We used Cox proportional hazards regression adjusted for condom use to compare pregnancy incidence during both perfect and typical (i.e., allowing temporary interruptions) use.

**Results:**

A total of 7710 women contributed to this analysis. Seventy pregnancies occurred during perfect and 85 during typical use. There was no statistically significant difference in perfect use pregnancy incidence among the methods: 0.61 per 100 woman-years for DMPA-IM [95% confidence interval (CI) 0.36–0.96], 1.06 for copper IUD (95% CI 0.72–1.50) and 0.63 for LNG implants (95% CI 0.39–0.96). Typical use pregnancy rates were also largely similar: 0.87 per 100 woman-years for DMPA-IM (95% CI 0.58–1.25), 1.11 for copper IUD (95% CI 0.77–1.54) and 0.63 for LNG implants (95% CI 0.39–0.96).

**Conclusions:**

In this randomized trial of highly effective contraceptive methods among African women, both perfect and typical use resulted in low pregnancy rates. Our findings provide strong justification for improving access to a broader range of longer-acting contraceptive options including LNG implants and copper IUD for African women.

**Implications statement:**

Data from this study support recommendations to providers, policy makers and patients that all of these methods provide safe and highly effective contraception for African women.

## 1 Introduction

Approximately 40% of the pregnancies that occur annually are unintended [[1], [2], [3]], making unintended pregnancy an issue of global public health importance [4,5]. Approximately 39% of women in sub-Saharan Africa report unintended pregnancies [6] [[1], [2], [3]]. These unintended pregnancies have substantial effects on both maternal and newborn health [[7], [8], [9]], completion of maternal education [10,11] and overall negative socioeconomic impacts on women and communities [8,12,13]. While many unintended pregnancies are due to lack of access to effective contraception, particularly to highly effective long-acting reversible methods, incorrect or inconsistent use and method failure are also important contributors [[14], [15], [16]].

Contraceptive failure rates can vary by body mass index, weight, age, education, socioeconomic status, contraceptive intention, residence and marital status [14,[17], [18], [19], [20]]. Highly effective long-acting reversible contraceptives that are not dependent on clients' ability to use them consistently and correctly — such as levonorgestrel (LNG) implants and intrauterine devices (IUDs) — generally have very low rates of contraceptive failure [[21], [22], [23], [24], [25]]. In contrast, methods that require user action, such as intramuscular injectable depot medroxyprogesterone acetate (DMPA-IM), have higher failure rates with typical use [16] due to delayed repeat injections. Data from Africa on contraceptive effectiveness, obtained through prospective, rigorously conducted studies, are lacking [6,26,27], particularly for IUDs [28]. Despite having been introduced in sub-Saharan Africa long ago, the provision of IUDs by health care providers and use by women has been hampered by negative product publicity as well as provider- and community-level barriers [[29], [30], [31]]. Good quality data from Africa are necessary for the framing of contraceptive counseling messages and informing service delivery strategies, including increased access to long-acting reversible contraceptives, to enable women to make informed contraceptive choices.

We conducted the Evidence for Contraceptive Options and HIV Outcomes (ECHO) Trial, a large multicenter, open-label, randomized clinical trial whose primary objective was to compare HIV incidence among women randomized to DMPA-IM, a copper IUD and an LNG implant. A secondary objective of the trial was to compare pregnancy incidence rates among the randomized contraceptive methods [32].

## 2 Material and methods

Between December 2015 and September 2017, we enrolled 7829 sexually active women aged 16–35 years from four countries (eSwatini, Kenya, South Africa and Zambia). Inclusion criteria included the following: not desiring pregnancy for at least 18 months, desired effective contraception and willing to consent to being randomized to any of the three contraceptive methods. We used variable block randomization, stratified by site, to assign women in a 1:1:1 ratio to receive DMPA-IM (150 mg/1 mL, Depo Provera, Pfizer), copper IUD (Optima TCu380A, Injeflex) or LNG implant (Jadelle, Bayer). Women were followed up every 3 months for a maximum of 18 months. Ethics review committees associated with each site provided approval for the study; informed consent was obtained from each woman prior to commencement of study procedures. Detailed methods of the trial have been described previously [32,33].

Women received their assigned contraceptive methods on-site at enrollment. DMPA-IM injection was repeated on-site every 3 months (13–17 weeks). We confirmed LNG implant presence by palpation at every visit and copper IUD presence by pelvic exam at 1-month follow-up visit, the final visit and when clinically indicated. At each visit, we collected data on current contraceptive use, and if a participant had discontinued her randomized method, we recorded the date and reason for discontinuation. At enrollment and at the final study visit, women underwent urine pregnancy testing; at interim visits, pregnancy testing was done as needed based on clinical judgment (e.g., missed/late menstrual period) or other indications (late DMPA injection, IUD strings not visualized) or participant request. Women who became pregnant were referred for antenatal care services but continued to be followed up in the trial.

At baseline, we tested all women for sexually transmitted infections (STIs) (*Chlamydia trachomatis*, *Neisseria gonorrhoeae*) using both syndromic and etiologic diagnoses and provided treatment. During follow-up, we provided syndromic STI management as needed.

### 2.1 Assignment of timing of incident pregnancies

**Fig. 1:**
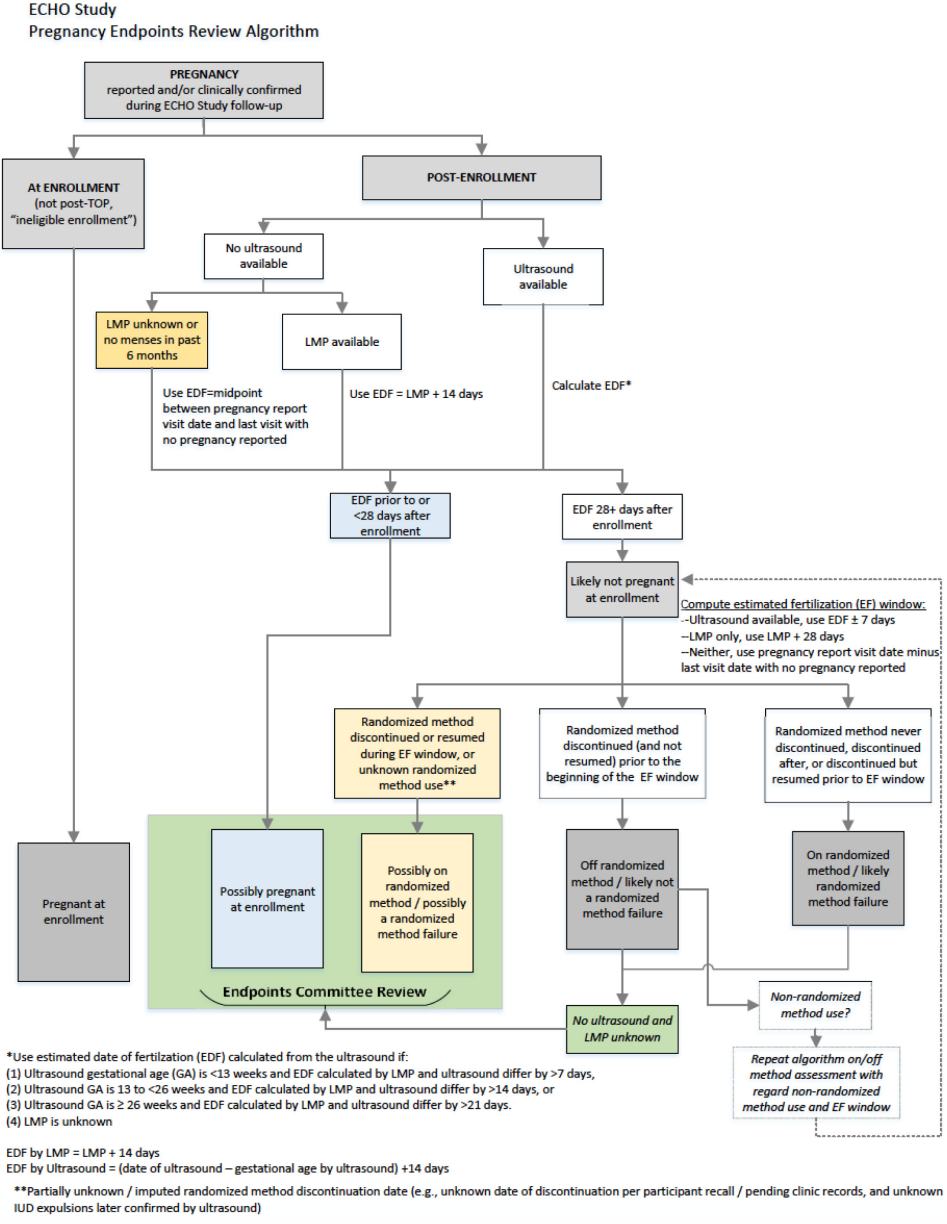
Pregnancy endpoints review algorithm.

Per a standard algorithm (Fig. 1), an estimated date of fertilization (EDF) was computed as the first day of last menstrual period (LMP) plus 14 days or, when available, ultrasound date minus gestational age, plus 14 days, with specific guidelines for when priority was given to the ultrasound estimated EDF if both LMP and ultrasound were available. An ECHO trial Pregnancy Endpoints Review Committee blinded to randomization group and current contraceptive use reviewed all pregnancies with computed EDFs in close proximity to randomized method discontinuation, with no ultrasound or LMP available, or with missing or unknown data on randomized method use. We excluded pregnancies where the EDF was estimated to have occurred prior to the trial enrollment visit, reflecting early pregnancy not detected by the urine pregnancy test at the enrolment visit.

### 2.2 Statistical analysis

The principal objective of this analysis was to compare pregnancy incidence among those randomized and using DMPA-IM, a copper IUD and an LNG implant within a multicenter, open-label, randomized clinical trial. Results of intention-to-treat analyses of contraceptive method incident pregnancy (which includes women who never started their randomized method or failed to adhere to it) were presented in the primary trial findings [33]; however, the majority of pregnancies occurred among women who were no longer using their randomized method. The present analysis focuses on pregnancies occurring among women who were randomized, initiated and were continuing their assigned method.

We defined two categories of method use: perfect use and typical use. For perfect use, time on method included time from the date of randomized method initiation until first discontinuation of the randomized method, first pregnancy (first estimated date of fertilization occurring after randomized method initiation) or until the final study visit. For women assigned DMPA-IM, if more than 17 weeks had passed since the previous injection, they were considered to have discontinued their randomized method. For women in the copper IUD group, randomized method discontinuation was the date of the first IUD expulsion or removal, regardless of whether a new device was inserted. For women in the LNG implant group, discontinuation was the date of first implant removal unless reinserted the same day.

For typical use, time on method was similar to that for perfect use, except it allowed for method use interruptions, such as time off method due to temporary clinician-initiated holds, late DMPA injections, missing implant rods (unless known to have been removed) and IUD expulsions (if reinserted within 28 days). For all three groups under both perfect and typical use, if another contraceptive method was initiated, the randomized method was considered to have been discontinued. Use of condoms in addition to any contraceptive method (so-called dual method use) was encouraged for STI/HIV protection and did not count as initiation of “another contraceptive method.”

After review by the Pregnancy Endpoints Review Committee, we realized that, for many pregnancies, dates of method discontinuation or estimated fertilization were based on limited data. Thus, we conducted a sensitivity analysis via an unblinded review of 47 pregnancies classified as “on method” in the perfect use analysis for which additional narrative notes were available from study clinical records. Two members of the endpoints committee made independent determinations of “very likely,” “likely,” “unlikely” or “very unlikely” that the pregnancy truly occurred while on the randomized method and then reached consensus on discrepant decisions; when there was a lack of consensus, a third member of the committee reviewed and acted as a tiebreaker. Determinations of “unlikely” or “very unlikely” were reclassified as nonevents and censored 1 week prior to the EDF for purposes of this sensitivity analysis.

Descriptive statistics were used to summarize participant characteristics at enrollment. We calculated the number of incident pregnancies, women-years at risk of pregnancy, crude pregnancy rates and exact 95% confidence intervals (CIs) for pregnancy rates based on a Poisson distribution, overall and within prespecified subgroups, for each randomized group. We estimated the cumulative probability of pregnancy using Kaplan–Meier methods, with 95% CIs based on the complementary log–log transformation. Cox proportional hazards regression model with a three-way class variable for randomized group, incorporating the baseline covariates (if significantly different between groups at p < 0.1) and stratified by site, was used to assess differences in pregnancy incidence between randomized groups: DMPA-IM vs. copper IUD, DMPA-IM vs. LNG implant and copper IUD vs. LNG implant. Standard errors for the parameter estimates from the Cox model were used to calculate *Z*-scores against the null hypothesis of hazard ratio (HR) = 1.0 and calculate corresponding two-sided p values; all tests were two-sided at the .05 significance level. We used SAS version 9.4 for all analyses.

## 3 Results

### 3.1 Study participants

**Fig. 2:**
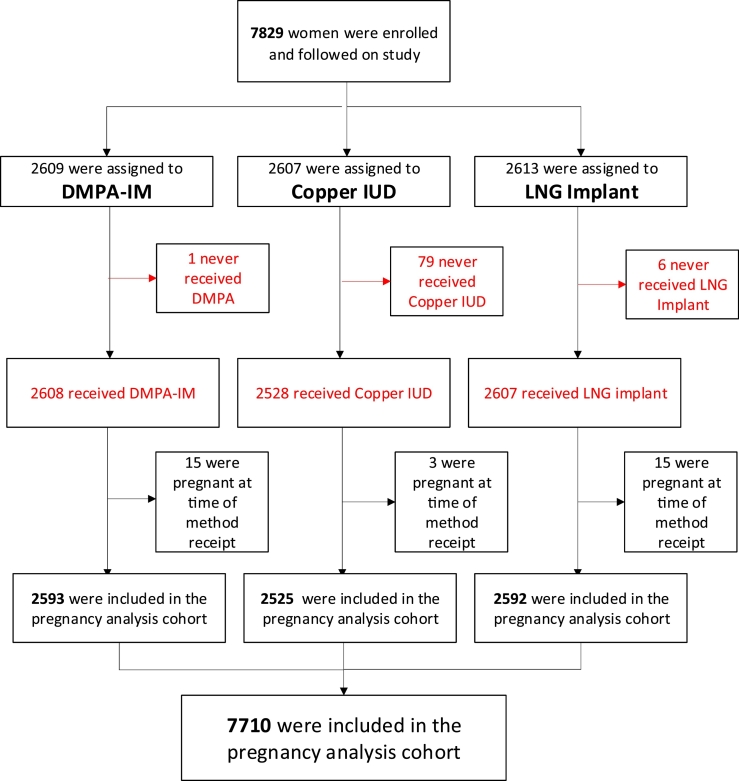
Summary of pregnancy analysis cohort.

A total of 7829 women were enrolled and randomized in the study. Of these, 7710 received their randomized method and were determined not to be pregnant at the time of randomization (Fig. 2). Baseline demographics and behavioral data were similar across randomization groups (Table 1). The median age was 23 years. The majority of participants were single and never married (79.9%), were not living with a partner (69.7%), had some or complete secondary school education (74.3%), owned a mobile phone (93.1%), had a body mass index (BMI) ≤ 30 kg/m^2^ (74.1%), and had one to two living children (66.2%). About half of the women did not use a condom during the last vaginal sex act (48.3%) and about half had ever used DMPA-IM (51.0), while 0.8% had previously used an IUD and 6.4% an implant. The prevalence of sexually transmitted infections at screening was high, with 18.1% having *Chlamydia trachomatis* and 4.7% having *Neisseria gonorrhoeae*.

**Table 1:** Demographic characteristics by randomized arm.

		DMPA-IM (*N* enrolled = 2593)	Copper IUD (*N* enrolled = 2525)	LNG implant (*N* enrolled = 2592)	All (*N* enrolled = 7710)
**Characteristic**	**Category**				
Age (years)					
Age group (years)	Median (IQR)	23 (20–26)	23 (20–26)	23 (20–26)	23 (20–26)
	16–17	17 (0.7%)	26 (1.0%)	21 (0.8%)	64 (0.8%)
	18–20	692 (26.7%)	656 (26.0%)	676 (26.1%)	2024 (26.3%)
	21–24	947 (36.5%)	882 (34.9%)	947 (36.5%)	2776 (36.0%)
	25–30	715 (27.6%)	737 (29.2%)	732 (28.2%)	2184 (28.3%)
	31–35	222 (8.6%)	224 (8.9%)	216 (8.3%)	662 (8.6%)
Marital status	Never married	2074 (80.0%)	2018 (79.9%)	2068 (79.8%)	6160 (79.9%)
	Married	499 (19.2%)	495 (19.6%)	499 (19.3%)	1493 (19.4%)
	Previously married	20 (0.8%)	12 (0.5%)	25 (1.0%)	57 (0.7%)
Lives with partner	Yes	759 (29.3%)	760 (30.1%)	755 (29.1%)	2274 (29.5%)
	No	1815 (70.0%)	1746 (69.1%)	1812 (69.9%)	5373 (69.7%)
	N/A, no partner	19 (0.7%)	19 (0.8%)	25 (1.0%)	63 (0.8%)
Education	No schooling	16 (0.6%)	12 (0.5%)	21 (0.8%)	49 (0.6%)
	Primary school, some or complete	215 (8.3%)	244 (9.7%)	257 (9.9%)	716 (9.3%)
	Secondary school, some or complete	1956 (75.4%)	1866 (73.9%)	1906 (73.5%)	5728 (74.3%)
	Postsecondary school	406 (15.7%)	403 (16.0%)	408 (15.7%)	1217 (15.8%)

Socioeconomic status					
Owns a mobile phone	Yes	2420 (93.3%)	2359 (93.4%)	2397 (92.5%)	7176 (93.1%)
Earns an income of her own	Yes	563 (21.7%)	557 (22.1%)	561 (21.6%)	1681 (21.8%)
BMI (kg/m^2^)	≤ 30	1942 (74.9%)	1883 (74.6%)	1890 (72.9%)	5715 (74.1%)
	> 30	645 (24.9%)	641 (25.4%)	696 (26.9%)	1982 (25.7%)
Ever contraceptive use^a^	IUD	18 (0.7%)	20 (0.8%)	20 (0.8%)	58 (0.8%)
	Implant	164 (6.3%)	167 (6.6%)	163 (6.3%)	494 (6.4%)
	DMPA	1288 (49.7%)	1313 (52.0%)	1332 (51.4%)	3933 (51.0%)
	Other hormonal method^b^	837 (32.3%)	819 (32.4%)	818 (31.6%)	2474 (32.1%)
	Other nonhormonal method^b^	1505 (58.0%)	1464 (58.0%)	1496 (57.7%)	4465 (57.9%)
	Other method	28 (1.1%)	19 (0.8%)	30 (1.2%)	77 (1.0%)
Number of living children	0	590 (22.8%)	532 (21.1%)	551 (21.3%)	1673 (21.7%)
	1–2	1700 (65.6%)	1673 (66.3%)	1733 (66.9%)	5106 (66.2%)
	≥ 3	303 (11.7%)	320 (12.7%)	308 (11.9%)	931 (12.1%)
Condom use with last vaginal sex	No	1222 (47.1%)	1240 (49.1%)	1259 (48.6%)	3721 (48.3%)
	Yes	1281 (49.4%)	1192 (47.2%)	1244 (48.0%)	3717 (48.2%)
	Partner, no sex	83 (3.2%)	86 (3.4%)	81 (3.1%)	250 (3.2%)
	No partner	5 (0.2%)	7 (0.3%)	8 (0.3%)	20 (0.3%)

*Sexually transmitted infections*					
*C. trachomatis*	Negative	2133 (82.3%)	2055 (81.4%)	2110 (81.4%)	6298 (81.7%)
	Positive	452 (17.4%)	469 (18.6%)	478 (18.4%)	1399 (18.1%)
	Not done	1 (0.0%)	0 (0.0%)	1 (0.0%)	2 (0.0%)
*N. gonorrhoeae*	Negative	2471 (95.3%)	2404 (95.2%)	2462 (95.0%)	7337 (95.2%)
	Positive	115 (4.4%)	120 (4.8%)	126 (4.9%)	361 (4.7%)
	Not done	0 (0.0%)	0 (0.0%)	1 (0.0%)	1 (0.0%)

a More than one contraceptive method may be reported.

b Other hormonal method includes norethisterone enanthate (NET-EN), oral contraceptives, patch and intravaginal ring. Other non-hormonal method includes male/female condoms, diaphragm/sponge, other barrier method, spermicide alone, natural methods such as withdrawal or rhythm method and tubal ligation, hysterectomy or other surgical sterilization.

### 3.2 Follow-up and contraceptive method continuation

Of the 7710 women included in this analysis, 7608 (98.7%) initiated their randomized method at enrolment. The remaining 102 women had delayed randomized method initiation (98 in copper IUD group and 4 in LNG implant group); the main reasons for not receiving randomized method were difficult insertions of IUD or implant or postponed insertions of IUD due the presence of symptomatic cervical infection. In cases of delayed method initiation, the median number of days from randomization to actual receipt of the method was 6 days for both copper IUD [interquartile range (IQR) 2–12] and LNG implant (IQR 2–13). Participants contributed a total of 9249 woman-years of follow-up to the perfect use analysis and 9853 woman-years to the typical use analysis.

### 3.3 Pregnancy incidence by randomized arm

A total of 70 incident pregnancies occurred during perfect use: 18 among women assigned DMPA-IM, 31 copper IUD and 21 LNG implant (Table 2). Overall, pregnancy incidence rates during perfect use were 0.76 per 100 woman-years (95% CI 0.59–0.96): 0.61 for DMPA-IM (95% CI 0.36–0.96), 1.06 for copper IUD (95% CI 0.72–1.50) and 0.63 for LNG implants (95% CI 0.39–0.96). Adjusted hazard ratios (aHRs) for pregnancy during perfect use were 0.56 (95% CI 0.32–1.01, p = 0 .053) for DMPA-IM compared with copper IUD, 0.93 (95% CI 0.50–1.75, p = .83) for DMPA-IM compared with LNG implant and 1.65 (95% CI 0.95–2.88, p = 0.08) for copper IUD compared with LNG implant.

**Table 2:** Statistical comparisons of pregnancy incidence by randomized group

	DMPA-IM			Copper IUD			LNG implant			DMPA-IM vs. copper IUD		DMPA-IM vs. LNG implant		Copper IUD vs. LNG implant	
	*N*	*N* events	Rate (95% CI)^a^	*N*	*N* events	Rate (95% CI)^a^	*N*	*N* events	Rate (95% CI)^a^	aHR^a^ (95% CI)^b^	p value	aHR^a^ (95% CI)^b^	p value	aHR^a^ (95% CI)^b^	p value
Perfect use	2593	18	0.61 (0.36–0.96)	2525	31	1.06 (0.72–1.50)	2592	21	0.63 (0.39–0.96)	0.56 (0.32–1.01)	.053	0.93 (0.50–1.75)	.827	1.65 (0.95–2.88)	.075
Unblinded perfect use	2593	15	0.50 (0.28–0.83)	2525	23	0.79 (0.50–1.18)	2592	18	0.54 (0.32–0.85)	0.63 (0.33–1.21)	.167	0.91 (0.46–1.81)	.793	1.44 (0.78–2.68)	.245
Typical use	2593	29	0.87 (0.58–1.25)	2525	35	1.11 (0.77–1.54)	2592	21	0.63 (0.39–0.96)	0.80 (0.49–1.31)	.375	1.39 (0.80–2.45)	.246	1.74 (1.01–2.99)	.044

Follow-up time was computed as time from first randomized method initiation to the first of method discontinuation, pregnancy or last clinic visit with no pregnancy up to the 18-month follow-up period.

a Adjusted for no condom use with last vaginal sex, which was the only baseline cofactor found to be associated with time to preganancy at p < 0.1. Individual Cox proportional hazards model results for this co-factor for perfect use: HR 1.73 (95% CI 1.06–2.83), typical use: HR 1.72, 95% CI 1.10–2.67.

b Exact 95% CI for incidence rate computed using the Poisson distribution.

After unblinded review, 56 of 70 pregnancies were determined to have likely or very likely occurred during perfect use, while the remaining 14 were likely off method. In this sensitivity analysis, perfect use pregnancy incidence rates were 0.50 for DMPA-IM (95% CI 0.28–0.83), 0.79 for copper IUD (95% CI 0.50–1.18) and 0.54 for LNG implant (95% CI 0.32–0.85). aHRs for pregnancy during perfect use in this sensitivity analysis were 0.63 (95% CI 0.33–1.21, p = 0·17) for DMPA-IM compared with copper IUD, 0.91 (95% CI 0.46–1.81, p = 0.79) for DMPA-IM compared with LNG implant and 1.44 (95% CI 0.78–2.68, p = 0.24) for copper IUD compared with LNG implant.

Eighty-five pregnancies were observed during typical use (29 among women assigned DMPA-IM, 35 copper IUD and 21 LNG implant). Typical use pregnancy incidence rates were 0.86 per 100 woman-years (95% CI 0.69–1.07): 0.87 for DMPA-IM (95% CI 0.58–1.25), 1.11 for copper IUD (95% CI 0.77–1.54) and 0.63 for LNG implant (95% CI 0.39–0.96). In typical use analysis, copper IUD was associated with a marginally statistically significant higher risk of pregnancy compared to LNG implant (aHR 1.74, 95% CI 1.01–2.99, p = 0.04). The other comparisons did not reach statistical significance: 0.80 (95% CI 0.49–1.31, p = 0.37) for DMPA-IM compared with copper IUD and 1.39 (95% CI 0.80–2.45, p = 0.25) for DMPA-IM compared with LNG implant (Table 2).

**Fig. 3:**
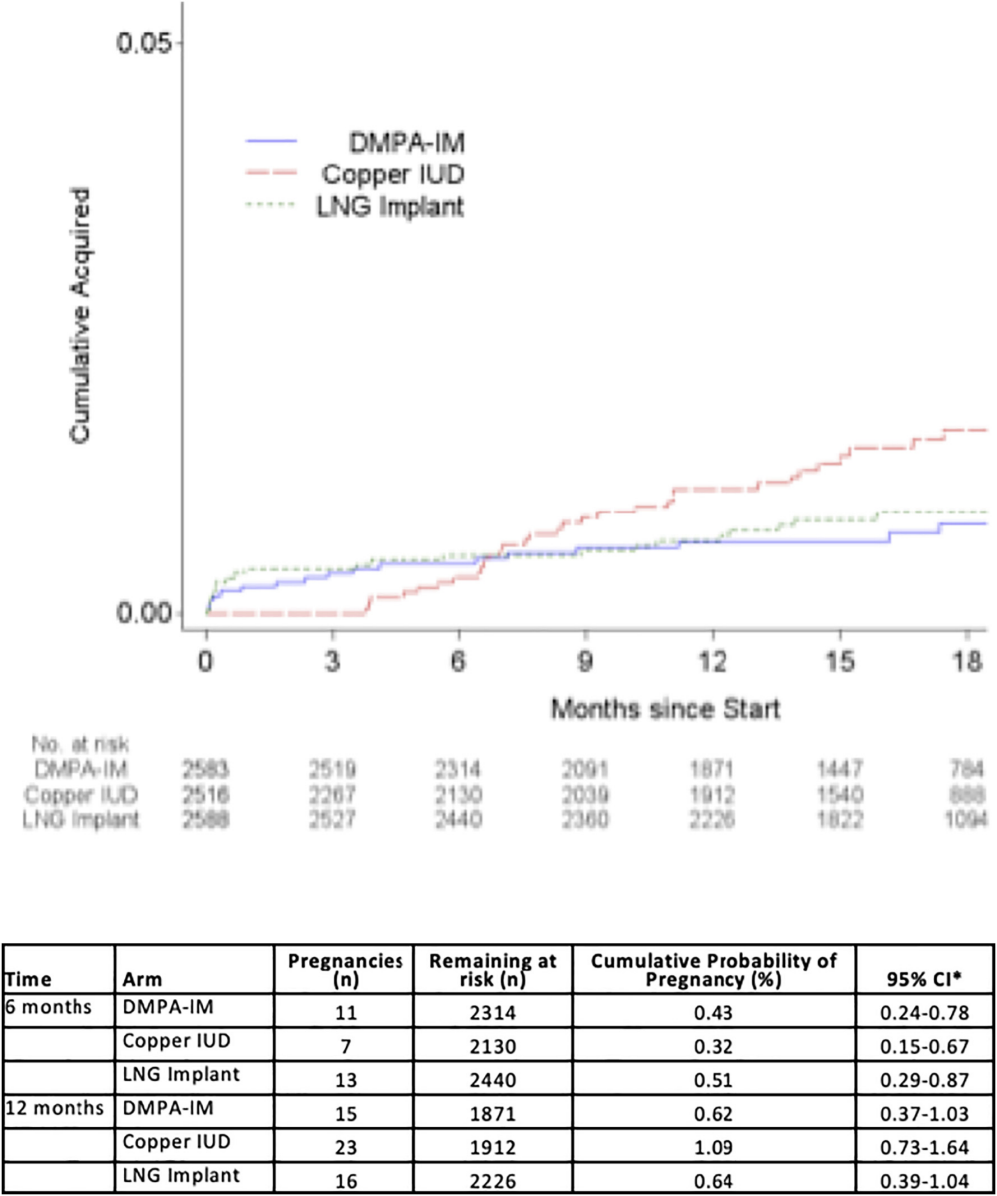
Perfect use pregnancy cumulative incidence (5% scale). *95% CI for cumulative probability computed from pointwise CIs for the survival function (Kaplan–Meier estimates).

**Fig. 4:**
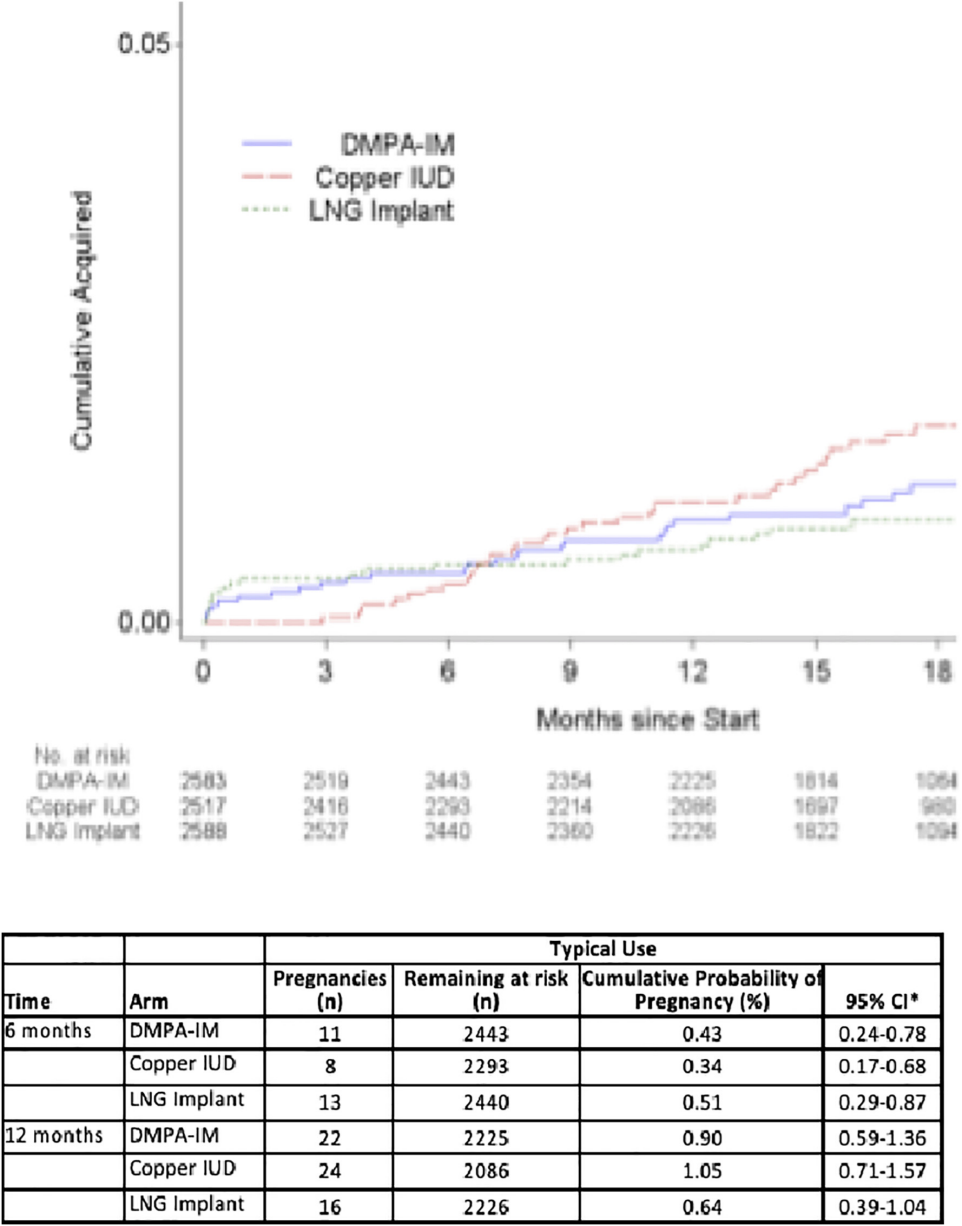
Pregnancy cumulative incidence: typical use. *95% CI for cumulative probability computed from pointwise CIs for the survival function (Kaplan–Meier estimates).

The cumulative probability of pregnancy at 6 and 12 months was low and similar in both perfect and typical use. Cumulative probability of pregnancy at 6 months with perfect use was 0.43% (95% CI 0.24–0.78) for DMPA-IM, 0.32% (95% CI 0.15–0.67) for the copper IUD and 0.51% (95% CI 0.29–0.87) for the LNG implant and was similar to the cumulative probability with typical use for the same period. Cumulative probability of pregnancy at 12 months with perfect use was 0.62% (95% CI 0.37–1.03) for DMPA-IM, 1.09% (95% CI 0.73–1.64) for copper IUD and 0.64% (95% CI 0.39–1.04) for the LNG implant. The cumulative probabilities with typical use at 12 months were similarly low (Fig. 3, Fig. 4).

## 4 Discussion

In this analysis of pregnancy rates among African women randomized to DMPA-IM, a copper IUD or an LNG implant, pregnancy incidence was low in all three groups with both perfect and typical use. Women using a copper IUD had slightly higher pregnancy rates than those using LNG implants or DMPA-IM, but absolute differences in rates were small.

The ECHO trial 12-month cumulative pregnancy rates for all three methods are similar or somewhat lower than those in a 2016 study [17] that analyzed contraceptive failure rates in 43 developing countries. That study analyzed Demographic and Health Survey Data in 43 countries and also found that the 12-month pregnancy rates for IUD users in developing countries were generally somewhat higher than commonly reported rates from US studies, varying from 0.9% to 2.2% (with an overall average of 1.4%). Higher rates may be due to a combination of factors, one being the fact that relatively few providers in sub-Saharan Africa have been trained to proficiently insert IUDs, and due to low IUD demand, even fewer are able to perform enough insertions to maintain their skills after training [34]. Limited provider experience with IUD insertions may lead to a higher rate of expulsions, while timely diagnosis of IUD expulsion is often not possible: complete expulsions may occur without women noticing, and partial expulsions often remain asymptomatic and diagnosed only by accident (e.g., when pelvic exam is done for some other reason) [29]. In spite of that, typical use pregnancy incidence of 1.11 among IUD users in ECHO was not higher than US-based pregnancy incidence rates of 0.8 per 100 woman-years [25], and thus, the copper IUD remained a highly effective method in our study.

Of note, we found very little difference between perfect and typical pregnancy incidence among DMPA-IM users, with typical use rates being much lower than those reported in the literature. This may be due to the rigorous manner in which participants in the ECHO Trial were followed and reminded to return for reinjection to the study clinic every 3 months, and the intensive contraceptive counseling and timely management of side effects. For long-acting reversible contraceptives (LARCs) such as implants and copper IUD, typical use rates are conventionally not separated from perfect use, as these methods are generally not considered to be user dependent [16]. Nevertheless, our typical use analysis adds value as it indicates what pregnancy rates might be in a program when IUD users may experience expulsions and reinsertions, or when use of IUD or implant might be temporarily stopped for various reasons (e.g., infection) but resumed when the issue is resolved. It also suggests that good counseling and proactive follow-up strategy can improve outcomes for shorter-acting methods as demonstrated by the similar typical and perfect use pregnancy incidences for DMPA-IM.

The cumulative incidence curves for copper IUD in this study show a different trajectory from those of DMPA-IM and LNG implant: in the first 3 months after randomization, few pregnancies occurred for women assigned the copper IUD. Unlike DMPA-IM and LNG implant, once inserted, a copper IUD is immediately effective for preventing pregnancy and also confers emergency contraceptive benefits if the woman had unprotected sex in the 5 days prior to insertion. The subsequent increase in cumulative pregnancies likely represents a consequence of partial or complete expulsions. Because we followed existing standard of care, we did not routinely ascertain placement of the copper IUD beyond the month 1 visit; most IUD expulsions (complete or partial) over the course of ECHO trial were identified with delay. This implies that even though one of the benefits of LARC is the elimination of the need to see health care providers regularly, women should be advised to check for any changes in the length of the string; if longer or missing, that could indicate expulsion and the need to seek the health care provider. While follow-up visits beyond 1 month after insertion may not impact contraceptive continuation or correct use [35], these visits would provide health care workers and women not only the opportunity to confirm that the IUD is in place but also the opportunity to provide other services such as STI diagnosis and management, HIV testing and cervical cancer screening.

Our study had several strengths including that data were collected within a high-quality clinical trial in which retention of participants was high. To the best of our knowledge, this is one of the first trials to randomly assign women to different contraceptive methods with follow-up to observe pregnancy incidence and outcomes [36,37]. This minimizes the observational and subjective biases that commonly affect studies in which women self-select methods. However, the intense follow-up of participants, including efforts to minimize number of missed follow-up visits; the intensive contraceptive counseling; and having ready access to study clinical teams for active evaluation and management of side effects have likely inflated typical use effectiveness in case of DMPA-IM. As such, it should be interpreted with caution in settings where there is no system in place for reminding DMPA-IM users to return for reinjection on time or for active follow-up of DMPA-IM users who are late for reinjection. The lack of ascertainment of placement of IUD every 3 months, while in line with existing standards of care, may have led to delay in detecting complete and partial expulsions. However, our unblinded sensitivity analyses reviewed every pregnancy in the study against documented participant and clinician notes to verify if the pregnancy truly occurred when on method.

In conclusion, in this study of African women, we found very low pregnancy rates among users of DMPA-IM, copper IUD and LNG implant. Our findings reinforce the need for improving access to a wider range of effective contraceptive options for women in Africa including long-acting methods such as implants and IUD. Our data can inform governments and program managers when they make financial and strategic decisions regarding contraceptive method mix expansion to enable women to achieve their reproductive health goals.

## References

[1] World Contraceptive Day Coalition (WCD). Global perspectives on unplanned pregnancies: a framework for action. WCD; 2017.

[2] Singh S., Sedgh G., Hussain R. (2010). Unintended pregnancy: worldwide levels, trends, and outcomes. Stud Fam Plann.

[3] Sedgh G., Singh S., Hussain R. (2014). Intended and unintended pregnancies worldwide in 2012 and recent trends. Stud Fam Plann.

[4] Oringanje C, Meremikwu MM, Eko H, Esu E, Meremikwu A, Ehiri JE. Interventions for preventing unintended pregnancies among adolescents. The Cochrane database of systematic reviews. 2016;2:Cd005215. 10.1002/14651858.CD005215.pub3 PMC8730506 26839116

[5] Bearak J., Popinchalk A., Alkema L., Sedgh G. (2018). Global, regional, and subregional trends in unintended pregnancy and its outcomes from 1990 to 2014: estimates from a Bayesian hierarchical model. Lancet Glob Health.

[6] Alkema L, Kantorova V, Menozzi C, Biddlecom A. National, regional, and global rates and trends in contraceptive prevalence and unmet need for family planning between 1990 and 2015: a systematic and comprehensive analysis. Lancet (London, England). 2013;381:1642–52. 10.1016/S0140-6736(12)62204-1 23489750

[7] Tsegaye A.T., Mengistu M., Shimeka A. (2018). Prevalence of unintended pregnancy and associated factors among married women in west Belessa Woreda, Northwest Ethiopia, 2016. Reproductive health.

[8] Yazdkhasti M., Pourreza A., Pirak A., Abdi F. (2015). Unintended pregnancy and its adverse social and economic consequences on health system: a narrative review article. Iran J Public Health.

[9] Gipson J.D., Koenig M.A., Hindin M.J. (2008). The effects of unintended pregnancy on infant, child, and parental health: a review of the literature. Stud Fam Plann.

[10] Aransiola J.O., Asa S., Obinjuwa P., Olarewaju O., Ojo O.O., Fatusi A.O. (2013). Teachers’ perspectives on sexual and reproductive health interventions for in-school adolescents in Nigeria. Afr J Reprod Health.

[11] Kavanaugh ML, Kost K, Frohwirth L, Maddow-Zimet I, Gor V. Parents' experience of unintended childbearing: a qualitative study of factors that mitigate or exacerbate effects. Social science & medicine (1982). 2017;174:133–41. 10.1016/j.socscimed.2016.12.024 PMC5258712 28038432

[12] Sonfield A., Kost K., Gold R.B., Finer L.B. (2011). The public costs of births resulting from unintended pregnancies: national and state-level estimates. Perspect Sex Reprod Health.

[13] Anema A., Fielden S.J., Castleman T., Grede N., Heap A., Bloem M. (2014). Food security in the context of HIV: towards harmonized definitions and indicators. AIDS Behav.

[14] Bradley S.E.K., Polis C.B., Bankole A., Croft T. (2019). Global contraceptive failure rates: who is most at risk?. Stud Fam Plann.

[15] Singh S DJaAL. Adding it up: the costs and benefits of investing in sexual and reproductive health. In: Institute G, editor. New York: Guttmacher Institute; 2014.

[16] Trussell J AA, Micks E, Guthrie KA. Efficacy, safety, and personal considerations. In: Hatcher RA NA, Trussell J, Cwiak C, Cason P, Policar MS, Edelman A, Aiken ARA, Marrazzo J, Kowal D, editor. Contraceptive technology. 21 ed. New York, NY: Ayer Company Publishers, Inc.; 2018.

[17] Chelsea Polis S.E.K.B. (2016). Akinrinola Bankole, Tsuyoshi Onda, Trevor N. Croft and Susheela Singh. Contraceptive failure rates in the developing world: an analysis of demographic and health survey data in 43 countries. In: Institute G, editor.

[18] Williams R.L., Fortenberry J.D. (2013). Dual use of long-acting reversible contraceptives and condoms among adolescents. J Adolesc Health.

[19] Metcalfe A., Talavlikar R., du Prey B., Tough S.C. (2016). Exploring the relationship between socioeconomic factors, method of contraception and unintended pregnancy. Reproductive health..

[20] Lopez LM, Bernholc A, Chen M, et al. Hormonal contraceptives for contraception in overweight or obese women. The Cochrane database of systematic reviews. 2016:Cd008452. 10.1002/14651858.CD008452.pub4 PMC9063995 27537097

[21] Sundaram A., Vaughan B., Kost K. (2017). Contraceptive failure in the United States: estimates from the 2006–2010 National Survey of Family Growth. Perspect Sex Reprod Health.

[22] Winner B., Peipert J.F., Zhao Q. (2012). Effectiveness of long-acting reversible contraception. N Engl J Med.

[23] Parks C., Peipert J.F. (2016). Eliminating health disparities in unintended pregnancy with long-acting reversible contraception (LARC). Am J Obstet Gynecol.

[24] Diedrich JT, Klein DA, Peipert JF. Long-acting reversible contraception in adolescents: a systematic review and meta-analysis. American Journal of Obstetrics and Gynecology. 2017;216:364.e1-.e12. 10.1016/j.ajog.2016.12.024 28038902

[25] Aiken JTaARA. Contraceptive technology 21st edition. In: Kowal D, Hatcher RA, Nelson AL, et al., editors. New York: Ardent Media; 2018.

[26] Darroch JE, Singh S. Trends in contraceptive need and use in developing countries in 2003, 2008, and 2012: an analysis of national surveys. Lancet (London, England). 2013;381:1756–62. 10.1016/S0140-6736(13)60597-8 23683642

[27] Blanchard K., Bostrom A., Montgomery E. (2011). Contraception use and effectiveness among women in a trial of the diaphragm for HIV prevention. Contraception.

[28] Buhling K.J., Zite N.B., Lotke P., Black K. (2014). Worldwide use of intrauterine contraception: a review. Contraception.

[29] Robinson N., Moshabela M., Owusu-Ansah L., Kapungu C., Geller S. (2016). Barriers to intrauterine device uptake in a rural setting in Ghana. Health Care Women Int.

[30] Greene E., Stanback J. (2012). Old barriers need not apply: opening doors for new contraceptives in the developing world. Contraception.

[31] Bergin A., Tristan S., Terplan M., Gilliam M.L., Whitaker A.K. (2012). A missed opportunity for care: two-visit IUD insertion protocols inhibit placement. Contraception.

[32] Hofmeyr G.J., Morrison C.S., Baeten J.M. (2017). Rationale and design of a multi-center, open-label, randomised clinical trial comparing HIV incidence and contraceptive benefits in women using three commonly-used contraceptive methods (the ECHO study). Gates open research.

[33] HIV incidence among women using intramuscular depot medroxyprogesterone acetate, a copper intrauterine device, or a levonorgestrel implant for contraception: a randomised, multicentre, open-label trial. Lancet (London, England). 2019. 10.1016/S0140-6736(19)31288-7 PMC6675739 31204114

[34] Gutin S.A., Mlobeli R., Moss M., Buga G., Morroni C. (2011). Survey of knowledge, attitudes and practices surrounding the intrauterine device in South Africa. Contraception.

[35] Steenland M.W., Zapata L.B., Brahmi D., Marchbanks P.A., Curtis K.M. (2013). The effect of follow-up visits or contacts after contraceptive initiation on method continuation and correct use. Contraception.

[36] Hubacher D., Spector H., Monteith C., Chen P.L. (2018). Not seeking yet trying long-acting reversible contraception: a 24-month randomized trial on continuation, unintended pregnancy and satisfaction. Contraception.

[37] Hubacher D., Spector H., Monteith C., Chen P.L., Hart C. (2017). Long-acting reversible contraceptive acceptability and unintended pregnancy among women presenting for short-acting methods: a randomized patient preference trial. Am J Obstet Gynecol.

